# Exploring huntington's disease from a neurodevelopmental perspective

**DOI:** 10.7150/ijbs.124552

**Published:** 2026-01-01

**Authors:** Chunhui Huang, Xiao Zheng, Wei Li, Zaijun Zhang, Shihua Li, Xiao-Jiang Li, Mingdeng Rong, Sen Yan

**Affiliations:** 1 The Sixth Affiliated Hospital of Jinan University, Dongguan, 523710, China; State Key Laboratory of Bioactive Molecules and Druggability Assessment, Guangdong Basic Research Center of Excellence for Natural Bioactive Molecules and Discovery of Innovative Drugs, Guangdong Provincial Key Laboratory of Non-human Primate Research, Guangdong-Hong Kong-Macau Institute of CNS Regeneration, Jinan University, Guangzhou, 510632, China; Stomatological Hospital, School of Stomatology, Southern Medical University, Guangzhou, 510280, China.; 2 Institute of New Drug Research, College of Pharmacy, Jinan University, Guangzhou, 510632, China.; 3 Department of Neurology, Guangzhou Red Cross Hospital, Faculty of Medical Science, Jinan University, Guangzhou, Guangdong, 510235, China.

**Keywords:** Huntington's disease, HTT, mHTT, neurodevelopment, animal model, brain organoids, early intervention

## Abstract

Huntington's disease (HD) is a rare, inherited neurodegenerative disorder caused by mutations in the *huntingtin (HTT)* gene. The classic concept is that HD is a degenerative disease that primarily affects the striatum, caused by a gain-of-function mutant mHTT that kills neurons. However, increasing evidence suggests that the effects of mHTT on development may be an alternative view of HD. Therefore, we describe the importance of HTT for neurodevelopment and then summarize the effects of mHTT on neurodevelopment that have been revealed so far in different models. Importantly, we provide new insights into the use of different models to study HD development, and propose new therapeutic strategies for intervening in HD early in development to improve disease progression. Furthermore, we explore potential connections between neurodevelopmental abnormalities and neurodegenerative processes in HD. This review provides a systematic synthesis of current knowledge regarding HD development and pathogenesis.

## 1. Introduction

Huntington's disease (HD) is an incurable rare neurodegenerative disease caused by mutations in the *huntingtin* (*HTT*) gene. The mutated *HTT* (*mHTT*) gene causes an abnormal CAG expansion (usually > 35), which subsequently leads to an elongated polyglutamine (polyQ) repeat in the protein, which may be more prone to folding and aggregation 1, 2. The classic concept is that HD is a degenerative disease that mainly affects the striatum and is caused by the gain of function of mutant mHTT that kills neurons, including transcriptional dysregulation, neuronal excitotoxicity, mitochondrial dysfunction, oxidative stress, and autophagy disorders 3, 4. However, there is a bottleneck in the exploration of HD drugs targeting the above mechanisms. So far, only tetrabenazine and deuterated tetrabenazine have been approved for the treatment of chorea. Although gene therapy is considered to be an effective way to treat HD, it requires more time and effort to develop it 5, 6. Therefore, it is of great significance to find new mechanisms and explore new treatment strategies.

HTT is widely present in various tissues, especially expressed at high levels in the brain 7. The importance of WT HTT to neural development is self-evident. It plays a key physiological role in neural development, including axonal transport, synapse development, neural rosette structure formation, neuronal migration, and regulation of neural progenitor cells (NPCs) and neurogenesis 8-12. In mice, homozygous deletion of HTT is embryonic lethal 13, 14. In addition, brain-specific HTT inactivation can lead to progressive neuronal defects, and the reduction of HTT makes it impossible to maintain brain development 15, 16. The above studies all show that normal HTT is crucial for neurodevelopment.

**Figure 1:**
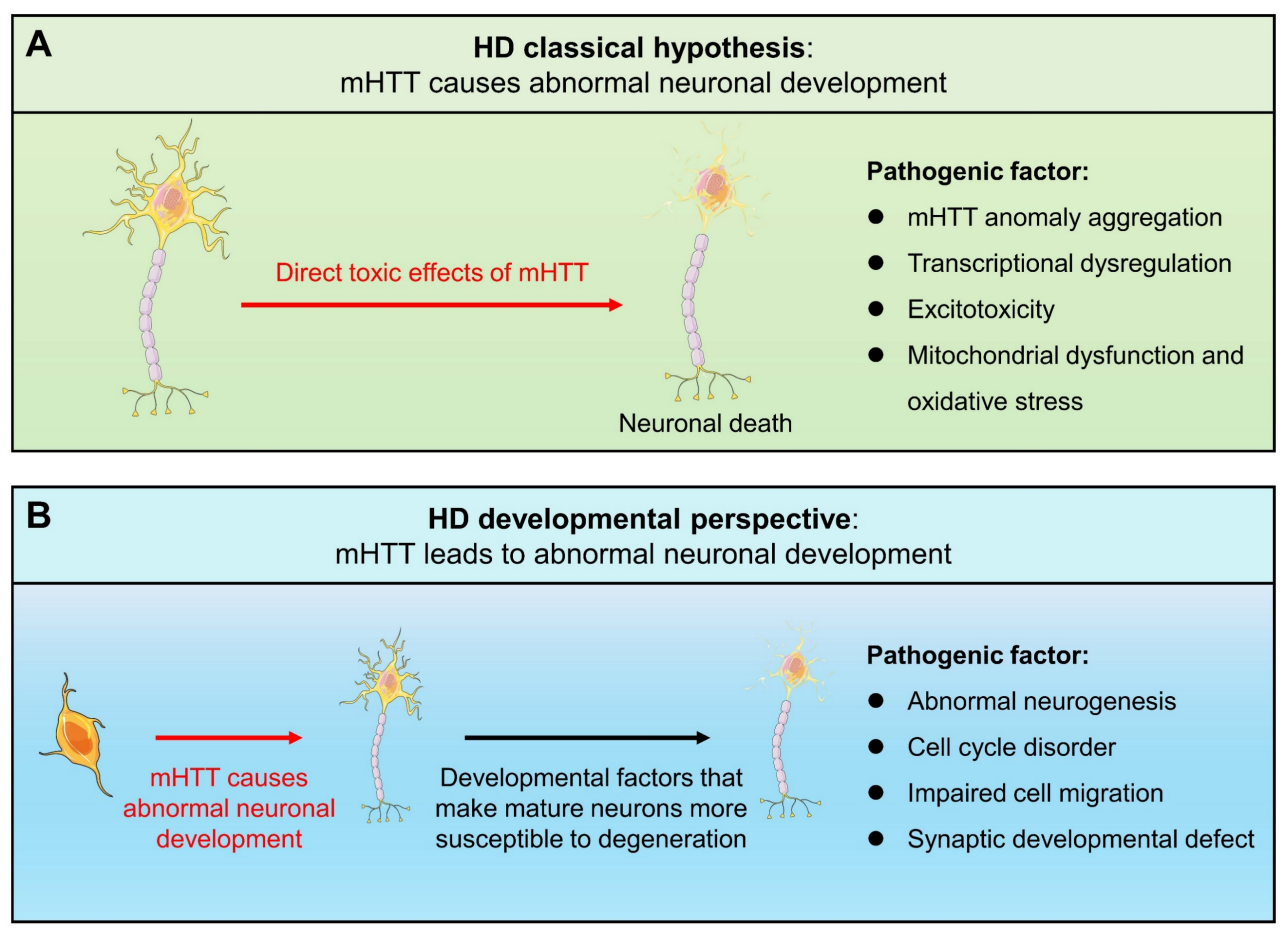
Schematic representation of classical pathogenic mechanisms and developmental views of HD.

Since mHTT expression is present throughout the life cycle, HD may have abnormal changes from the embryonic development process. A new view of HD disease is gradually emerging, that is, the impact of mHTT on the developmental level may be another mechanism causing HD (**Fig. 1**). Increasing evidence shows that the presence of mHTT does lead to abnormal neurodevelopment, including in human fetuses, animal models, stem cells, and brain organoids. When analyzing the changes in pathological and physiological mechanisms during HD development, potential origins of cellular susceptibility can be observed, leading to a deeper understanding of HD prodromal symptoms and different onset ages. Importantly, the mechanism of HD neurodevelopmental abnormalities provides a novel therapeutic window and strategy for early intervention and treatment of the disease, potentially delaying or mitigating disease progression. Therefore, it is necessary to explore the contribution of mHTT's effects on the developmental neural process to the disease, which will help us further understand HD and provide new ideas for exploring new treatment strategies for HD.

## 2. HTT is essential for embryonic and neurodevelopment

### 2.1. HTT is essential for embryonic development

HTT is critical in early embryonic development before the emergence of the nervous system. Genetic manipulations in mice have shown that HTT is essential for embryogenesis, craniofacial formation, and forebrain development 17. For example, homozygous deletion of the mouse homolog HD gene (*Hdh*) results in early embryonic lethality 13, 14, 18. This appears to be caused by increased apoptosis of cells in the embryonic ectoderm shortly after gastrulation, leading to embryonic death between 8.5 and 10.5 days. However, injection of embryonic stem (ES) cells lacking *Hdh* into wild-type host blastocysts averts early embryonic lethality in chimeric mouse embryos, which can develop and survive long after birth, indicating that the critical function of HTT in early development is in extraembryonic tissues 19.

Previous studies have identified multiple HTT-interacting proteins, such as Hap1, PSD95, and FIP-2, which play important roles in clathrin-mediated endocytosis, apoptosis, vesicle trafficking, cell signaling, morphogenesis, and transcriptional regulation, suggesting that HTT is also involved in these processes 20, 21. In addition, HTT is involved in vesicular trafficking, which plays an important role in the transport of nutrients across extraembryonic membranes, and disruption of this function may underlie the deleterious effects of *HTT* deletion on extraembryonic membranes 22-24. However, HD-causing mHTT does not disrupt this function, as HD patients with homozygous mutations do not exhibit embryonic lethality 25, 26. Moreover, mHTT, which expresses an abnormally expanded polyglutamine, can rescue *Hdh*-deficient mice from embryonic lethality 27, 28. In humans or mice, a single normal *HTT* allele is sufficient to ensure that development and postnatal life are largely or completely indistinguishable from normal life 13, 18. In summary, HTT is indispensable for the developing embryo, and HTT deficiency in the embryo is lethal.

### 2.2. HTT is an important component of neurodevelopment

HTT plays a key role in multiple stages of neurogenesis, including proliferation and differentiation of neural stem cells, neuronal migration, and synapse formation. Studies have shown that reduced levels of HTT expression lead to abnormal brain development and perinatal lethality, indicating that HTT is required for neurogenesis 29. Mice expressing very low levels of HTT from the embryonic period to 21 days after birth show late striatal and cortical neuronal degeneration, white matter tract damage, and axonal degeneration. These results suggest that developmental defects associated with HTT function make cells in specific neural lesions more susceptible to cell death 30.

Post-mortem studies of brains from individuals with HD have shown disorganized cortical layers, suggesting that neuronal migration was disrupted during development 31, 32. HTT localizes to the spindle poles during mitosis, and silencing of *HTT* in cells disrupts spindle orientation by mislocalizing dynactin, dynein, and the large nuclear mitotic apparatus NUMA1 (nuclear/mitotic apparatus protein 1) protein 12. Furthermore, specific disruption of HTT in neural cell precursors leads to poor spindles, while expression of HTT can restore spindle abnormalities. Knockdown of *HTT* in neocortical neuroepithelial cells results in disrupted cell migration, decreased proliferation, and increased cell death, whereas in the cerebellum, *HTT* knockdown results in cell death but does not interfere with migration, suggesting that the spatial and temporal requirements of HTT expression in neural development are relatively specific 11. The loss of HTT leads to abnormal position of neurons in specific layers of the mouse neocortex, especially in postmitotic projection neurons 12. HTT is essential for the transition of projection neurons from multipolar to bipolar and for maintaining the bipolar morphology during their radial migration 33.

Furthermore, HTT is required for normal excitatory synapse development in cortical and striatal circuits. When HTT function is silenced in the developing cortex, excitatory synapses in the cortex and striatum form and mature by postnatal day 21 (P21), followed by accelerated synapse loss accompanied by reactive gliosis 9. Another study showed that HTT coordinates the morphology and function of dendritic spines through cofilin-mediated control of the actin cytoskeleton, and cell-autonomous depletion of *HTT* leads to enlarged spines but reduced excitatory synaptic function 34.

## 3. mHTT causes abnormal neurodevelopment

### 3.1. Evidence from HD patients

#### 3.1.1. Defective brain structure development in presymptomatic HD gene carriers

Clinical studies of presymptomatic HD gene carriers have provided clues to the developmental disorders of HD (**Table 1**). Asymptomatic children at risk for HD had significantly reduced head circumference, weight, and BMI. Even after correction for height, head circumference remains abnormally low, suggesting a specific defect in brain development 35. MRI and PET can detect changes in the brains of HD gene carriers years before the disease appears 36. For example, neuroimaging scans of HD patient brains can detect changes in the volume of the striatum, cortex, and entire brain before symptoms appear. Specifically, atrophy of the caudate nucleus, putamen, globus pallidus, basal ganglia, and white matter are abnormal before HD symptoms 37. Another study similarly demonstrated region-specific gray and white matter atrophy in presymptomatic HD that correlated with early clinical phenotypes 38. Large neuropathological series have demonstrated an increased incidence of developmental malformations in the HD brain 39. Sulcal morphometry revealed the absence of asymmetry in the length of the sylvian imprint in HD, a profound asymmetry of this cortical region that is typically observed in healthy development 40. This finding suggests that HD is intertwined with neurodevelopment.

Recent studies have linked development and neurodegeneration through the genetic topology of HD and cortical cell loss 41. Cortical cell loss is a core feature of HD and begins in the presymptomatic stage many years before clinical motor diagnosis. Using volumetric and diffusion MRI, the researchers extracted specific whole-brain maps from 80 participants with an average age of 15 years before HD onset and 71 controls and found that lower gray matter volume and higher mean gray matter diffusivity could be used as a proxy for cortical cell loss in HD. Importantly, cortical cell loss was positively correlated with the expression of developmental genes and negatively correlated with the expression of synaptic and metabolic genes associated with neurodegenerative diseases. Therefore, cortical cell loss in the pre-HD stage may be caused by a dual pathological process, where the result of neurodevelopmental changes at the beginning of life and neurodegeneration in adulthood jointly lead to the onset of HD.

MRI was performed on children and adolescents whose parents or grandparents were diagnosed with HD, including 75 individuals who were gene-expanded (GE) and 97 individuals who were gene-nonexpanded (GNE). The GE group was estimated to have been clinically diagnosed for an average of 35 years. Results showed that GE initially presented with striatal and pallidum hypertrophy and had a more rapid rate of volume decline 42. Furthermore, when CAG>50, the extended length of CAG exaggerated this pattern. Similarly, the researchers used the Kids-HD and Kids-JOHD databases to investigate 7 children with CAG>50 who did not have motor manifestations of HD, and also found that the average striatal volume in the GE group was significantly higher than that in the GNE group 43. Continuous testing of one of the individuals with a CAG of 73 revealed that while this individual showed striatal hypertrophy similar to the GNE group until the age of six, this was reduced by nearly 20% approximately two years later.

#### 3.1.2. Cognitive and emotional abnormalities precede motor symptoms in HD

Clinical diagnosis of HD is usually made when motor symptoms and chorea appear, whereas cognitive and psychiatric symptoms of HD appear before motor manifestations. Psychiatric symptoms occur more frequently in the HD prodrome than previously thought 44. Atrophy of the caudate, putamen, globus pallidus, basal ganglia, and white matter was associated with subtle motor and cognitive measures early in the pre-HD phase 37. In addition, pre-HD show striatal atrophy and increased clinical irritability scores, which provides insights into the pathophysiology of early neuropsychiatric symptoms in pre-HD 45. Subtle HD-mediated changes occur decades before diagnosis, including disturbances in network topology and functional connectivity 46. Evaluation of executive function in children, adolescents, and young adults with GE has revealed significant deficits in comprehensive measures of executive function and working memory, and these deficits occur as early as 18 years of age, well before the motor manifestations of HD 47.

#### 3.1.3. Abnormal cortical development in HD fetuses

As mentioned above, imaging studies and presymptomatic features describe signs of HD before clinical diagnosis, which can be traced back to the developmental period. Emerging evidence strongly supports developmental abnormalities in HD, for example, an increased incidence of developmental malformations can be found in HD autopsy brain samples 39. Study in pre-HD show striatal atrophy and increased clinical irritability scores, which provides insights into the pathophysiology of early neuropsychiatric symptoms in pre-HD 45. More importantly, studies of HD fetuses during pregnancy have demonstrated that mHTT alters cortical development, which is the most direct evidence of developmental abnormalities in HD 48. This study demonstrated multifaceted developmental changes in HD, such as mislocalization of mHTT and junctional complex proteins, abnormalities in polarity and differentiation of neural progenitor cells, defects in cilia generation, and changes in mitosis and cell cycle. Specifically, the number of proliferating cells is reduced in HD, and more neural progenitor cells prematurely enter the neurogenic lineage specification.

### 3.2. Abnormal neurodevelopment in HD animal models

Although brain structural changes in presymptomatic HD gene-carrying adolescents and abnormal cortical development in HD fetuses provide strong support for the HD development hypothesis, how mHTT affects the neurodevelopmental process and the impact of HD developmental abnormalities on later disease progression require more mechanistic exploration. Currently, there is an increasing number of studies exploring the developmental mechanisms of HD through HD animal models (**Table 2**).

#### 3.2.1. Abnormal neurogenesis

In order to study the changes in brain microstructure in developing transgenic HD rat pups, it was observed that the average and radial diffusivity of white matter and gray matter structures in HD pups were higher and decreased with age, accompanied by an increase in fractional anisotropy in white matter structures 49. This suggests that abnormal neurodevelopment in HD rats changes the early brain structure. Previously, it was reported that hippocampal neurogenesis was reduced in both R6/2 and R6/1 mice, especially in the dentate gyrus 50-52. The olfactory bulb is one of the regions where subventricular zone (SVZ)-derived neuroblasts are derived. In the R6/2 HD mouse model, the adverse microenvironment associated with HTT protein in the olfactory bulb interferes with the survival and integration of new mature neurons, resulting in impaired neurogenesis in the adult olfactory bulb 53. HD knock-in (*Hdh*-Q111) mice exhibit delayed acquisition of early striatal cytoarchitecture and aberrant expression of progressive markers of MSN neurogenesis 54. Similarly, the olfactory bulb neurogenesis was significantly reduced in the more clinically relevant transgenic rat model of late-onset HD (tgHD rats) 55. In addition, the number of neuroblasts in 11-week-old R6/2 mice was greatly reduced, and NeuroD1 expression was reduced in the hippocampus, suggesting that neurogenesis in R6/2 mice was impaired at the level of NeuroD1 56.

Due to differences in model construction and CAG repeat sequences, HD transgenic mice exhibit a relatively rapid disease progression. Adult R6/1 and R6/2 mice show reduced hippocampal neurogenesis, similarly, decreased olfactory bulb neurogenesis has been observed in HD rats. HD knock-in mice (Hdh-Q111) demonstrate delayed acquisition of early striatal cytoarchitecture, which may be attributed to the delayed cell cycle exit of striatal progenitor cells. Therefore, the brain regions affected by developmental impairments may vary across different HD animal models.

#### 3.2.2. Cell cycle abnormalities

In Drosophila, the mHTT/polyQ repeat sequence impairs brain development by inhibiting the cell cycle 57. It can destroy the nuclear pore complex and nuclear import of neuronal cells, resulting in abnormal nuclear import of cell cycle regulatory proteins E2F, Cyclin E and PCNA from the cytoplasm to the nucleus. In addition, *Hdh*-Q111 striatal progenitors display delayed cell cycle exit between E13.5-15.5, an expansion of the intermediate progenitor pool, and overexpression of the transcription factor Sox2 54. *Hdh*-Q111 neural stem cells (NSCs) also exhibit abnormal development, including impaired lineage restriction, reduced proliferation potential, enhanced late self-renewal, and dysregulated MSN subtype specification.

Moreover, mHTT causes mitotic spindles in improperly divided progenitor cells. In *Hdh*Q111/Q111 mice, mHTT caused mitotic spindle misorientation in dividing progenitor cells and reduced cortical thickness at embryonic days E14.5 and E16.5 58. Similarly, mHTT caused mitotic spindle misorientation in embryonic fibroblasts derived from these mice by altering the subunits of dynein, NUMA1, and dynactin. Similar to HD fetuses, mHTT protein in *Hdh*Q111 mice causes mitotic spindle misorientation and biases the fate of progenitor cells toward the neuronal lineage 48.

Overall, HD knock-in mice exhibit delayed cell cycle exit of progenitor cells, reduced proliferative potential, and a bias of progenitors toward the neuronal lineage, which may represent key mechanisms underlying subsequent neurodevelopmental abnormalities.

#### 3.2.3. Impaired cell migration

MHTT also interferes with the migration and maturation of post-mitotic neurons. During cortical development, HTT regulation is required for the multipolar-bipolar transition of projection neurons and for maintaining their bipolar shape during radial migration 33. In *HTT* conditional knockout mice, loss of HTT resulted in delayed cell migration, while re-expression of WT HTT was sufficient to reproduce the function of HTT in migration. In contrast, pathological mHTT was unable to compensate for the loss of HTT, indicating that HD pathological HTT has lost its ability to promote neuronal migration 33. R6/2 mice did not show any significant increase in progenitor proliferation nor differences in progenitor apoptosis, with migration of neuroblasts along the rostral migratory stream being significantly diminished 59. Thus, progenitor cells have an impaired migration in their route to the olfactory bulb, with accumulation of cells in the caudal rostral migratory stream that does not result from changes in cell death. Another HD mouse model also showed abnormal cell migration ability. Compared with WT cells, neural stem/progenitor cells (NSPCs) derived from the YAC128 SVZ showed higher proliferation and migration capacity, with more MAP2^+^ and synaptophysin^+^ cells 60.

#### 3.2.4. Synaptic development abnormalities

Early synaptic problems in excitatory cortical and striatal connections have been reported in HD. By conditionally silencing *HTT* in the developing mouse cortex, excitatory synapses in the cortex and striatum form and mature at postnatal day 21. However, at 5 weeks, the original exuberant synaptic connections disappeared in the cortex, accompanied by gliosis. Interestingly, similar to *HTT* conditional knockout mice, in the zQ175 mouse model of HD, there is excessive excitatory synapse formation and maturation in the cortex at P21, which disappears after 5 weeks 9. Thus, HTT is required for the correct establishment of cortical and striatal excitatory circuits, while the presence of mHTT leads to abnormalities in cortical and striatal excitatory circuits. Tamoxifen was administered to BACHD:CAG-Cre (ERT2) mice at postnatal day 21 to terminate mHTT expression. These conditional knockout mice eventually exhibited impairments similar to those of mice that express mHTT throughout their lives 61. This strongly suggests that presymptomatic developmental abnormalities may play an important role in HD pathogenesis and progression.

As symptoms develop and worsen, R6/2 mice develop a significant reduction in low-amplitude synaptic currents, accompanied by a significant decrease in presynaptic and postsynaptic markers. However, early changes in striatal glutamatergic input in the presymptomatic R6/2 model were manifested by large-amplitude synaptic events (>100 pA) occurring more frequently, which may lead to selective vulnerability of striatal medium spiny neurons 62.

In additional, prior to neuronal loss, *mHTT* gene carriers have a thinner corpus callosum 63. Growth of layer 2/3 neurons in HD from *Hdh*Q7/Q111 mice is restricted by defects in microtubule bundling within axonal growth cones, resulting in reduced axons traversing the corpus callosum 64. Proteomic analysis of growth cones revealed that NUMA1 is downregulated in HD. Inhibition of NUMA1 in WT cells reproduced the microtubule and axon growth defects of HD, whereas elevated NUMA1 levels restored microtubule organization and rescued axon growth. Thus, NUMA1 downregulation induces axon growth defects in HD. In another study, in the first week after birth, *Hdh*Q7/Q111 mice had low excitatory synaptic activity in cortical layer 2/3, low glutamatergic receptor expression, and exhibited sensorimotor deficits, but these deficits could be self-repaired and returned to normal levels in the second week 65, 66. It is worth mentioning that strengthening glutamatergic synaptic transmission with drugs in the neonatal period can rescue these neural defects and improve sensorimotor and cognitive functions of HD model mice in adulthood.

In summary, varying synaptic developmental phenotypes have been observed across different HD mouse models. R6/2 mice exhibit significant impairments in synaptic development, including markedly reduced synaptic currents and decreased expression of synaptic markers. Similarly, HdhQ7/Q111 HD mice show a significant reduction in axons crossing the corpus callosum, indicating defects in axonal development. In contrast, the zQ175 HD mouse model displays excessive excitatory synapse formation and maturation in the cortex. Therefore, more suitable HD animal models and more in-depth studies are required to elucidate synaptic developmental abnormalities in HD.

### 3.3. Abnormal neurodevelopment in HD *in vitro* models

Studies in animal models have shown that HTT is intricately involved in corticogenesis, and mHTT may affect this process. However, the role of mHTT in human corticogenesis has not been investigated. Due to the intrinsic differences in the progression and timing of cortical development between humans and mice, it is critical to study the developmental progression of HD using induced pluripotent stem cells (iPSCs) and even brain organoids (**Table 3**). Patient-derived iPSCs, somatic cells reprogrammed to a pluripotent state, provide an important resource for deciphering the underlying mechanisms of neurological diseases and also help explore new treatments 67. iPSCs can be differentiated into multipotent neural stem progenitors that generate mature neural subsets 68. It has been previously reported that HD patient-derived iPSCs display extended CAG-related phenotypes, which is an important basis for studying HD development 69.

Studies on HD iPSCs during development have been reviewed 70. For instance, differentiating iPSC lines from HD patients (180, 109, or 77 CAG repeats) to a cortical fate, HD iPSCs generated similar numbers of deep and upper layer cortical neurons in the same amount of time as control iPSCs (33, 21, or 18 CAG repeats). However, HD cortical cells showed marked differences in gene expression compared to controls, many of which overlapped with gene expression detected in motor cortex regions of postmortem HD brains. Transcriptomics showed delayed maturation and altered morphology of HD cortical neurons, which was corroborated by electrophysiological studies and neurite measurements. Not only is the average neurite length generally higher in controls than in HD neurons, but increases in CAG repeat length are directly correlated with decreased neurite length 71. The presence of mHTTEx1 in hiPSCs-derived neurons produced developmental alterations, such as changes in the appearance of synaptic proteins and a reduction in synaptic contacts, and led to a delay in the development of mature neuronal activity patterns 72.

Another study performed omics analysis of neural cultures from iPSCs from HD patients, revealing that genes in glutamate and GABA signaling, axon guidance, and calcium influx were all reduced in HD 73. One-third of the altered genes were included in pathways that regulate neuronal development and maturation. Among them, REST is the most highly activated upstream regulator in HD cells. REST is associated with the neurotoxicity of mHTT and the dysregulation of genes such as BDNF 74. At the same time, it is also a major regulator of neurogenesis and neurodevelopment, which promotes the self-renewal of neural stem cells or progenitor cells, but limits the generation and maturation of neurons 75. These results indicate that mHTT impairs neurodevelopmental pathways.

Furthermore, there are more and more studies using organoids to study the mechanisms of HD developmental processes, which can more closely simulate the developmental progression of HD. Cortical organoids formed using HD-derived iPSC lines show a possible link between mHTT and abnormal neural development in HD 76. Large CAG expansions lead to a complete failure of neural ectoderm acquisition, while cells carrying shorter CAG repeats show severe abnormalities in neural rosette formation and disruption of cellular architecture. In addition, control organoids overlap with mature human fetal cortex gene expression, whereas HD organoids have gene expression profiles associated with immature ventricular/SVZ.

Brain organoids carrying *mHTT* (70Q/70Q) showed reduced overall growth rate compared with WT organoids, which is consistent with the characteristics of neurodevelopmental disorders 77. Specifically, brain organoids carrying mHTT showed severe disruptions in cellular organization, including the lack of ventricular zone-like neurogenic areas. In addition, NPCs appeared to be particularly impaired, as evidenced by the reduced presence and spatial disorganization of SOX2- and FOXG1-positive NPC cells, as well as the marked disruption of the tight junction marker ZO1. Transcriptional analysis of brain organoids confirmed that the expression of progenitor markers was lower in 70Q/70Q organoids, while the vast majority of genes associated with the GO term “nervous system development” were also downregulated. Moreover, mHTT-induced dysregulation of protein coiled-coil-helix-coiled-coil-helix domain containing 2 (CHCHD2) led to abnormal mitochondrial morphological dynamics, which in turn impaired mitochondrial function and organization of early neural cells.

The latest study used iPSCs from HD patients to construct human cortical organoids (hCOs). HD-hCOs exhibit abnormal cortical development trajectories, including defects in the connection complex in the neural tube, delayed postmitotic neuronal maturation, dysregulation of the fate specification of cortical neuron subtypes, and abnormalities in early HD subcortical projections during corticogenesis 76. The polyQ assembly involved in endogenous mHTT may reduce the ability of the Golgi apparatus to recruit ARF1 in neural precursor cells, thereby leading to abnormal cortical development in HD-hCOs 78.

Subsequent studies using single-cell RNA sequencing revealed neurodevelopmental abnormalities in ventral fate acquisition in HD telencephalic organoids, such as cytoarchitecture and transcriptional defects that result in a reduction in GABAergic neurons 79.

## 4. The need for appropriate animal models to study neurodevelopment in HD

A series of reports have highlighted the increase in SVZ progenitor populations in HD embryonic stem cells, mouse models, and human tissues, indicating upregulation of progenitor proliferation and neurogenesis, as well as abnormal neuronal migration and synaptic development defects during development. Although all conclusions support the existence of abnormalities in HD development that contribute to late disease progression, there are still differences in different models. For example, the HD cell cycle is inhibited in *Drosophila*, while delayed cell cycle exit of striatal progenitors in the Q111 mouse model leads to an expansion of the intermediate progenitor pool and overexpression of the transcription factor Sox2, which cannot be fully matched with fetal cell cycle disorders in human HD 48, 54, 57. In addition, studies have shown that HD has developmental defects in axons and synapses, such as low excitatory synaptic activity and reduced expression of glutamate receptors in cortical layer 2/3 in early postnatal HD mice 65. In cortical organoids, glutamate and GABA signaling gene expression is reduced and axon guidance is weakened 73. However, in the early stages of HD, autopsy brain slices show evidence of proliferative changes, such as increased number and size of dendritic spines, proliferation and size of SVZ, and increased number of newborn neurons 80-82. Furthermore, in mouse embryonic stem cells, those with the expanded repeat sequence had more elaborate and longer processes than those with normal CAG repeat sequence length, and the neurites in adult HD neurons were actually longer, suggesting that the maturation process of neurons is altered 83.

Animal models are essential for studying pathogenesis and developing treatments for human diseases. The reason for the same conclusion but different phenomena may be due to different models. HD rodent models vary greatly, including different CAG repeat sequence lengths and different model construction methods. In organoid models, iPSCs from different patients may be the main difference, including but not limited to culture conditions, induced organoid types, and detection time. Limited by the ethical limitations of human research, it is particularly important to have a suitable animal model to study the development of HD. Due to the closeness of the brain structure and size of large animals to humans, recent studies using CRISPR/Cas9 in large animals (pigs and monkeys) have discovered important pathological features of HD disease 84, 85. These events are similar to neurodegeneration in the brains of patients, reflecting the advantages of large animal models in studying diseases.

Shang-Hsun Yang *et al.* established a transgenic rhesus monkey model of HD, in which the hallmark features of HD, including nuclear inclusions and neuronal cell aggregates, were observed in the brain of this monkey model 86. In addition, transgenic monkeys show important clinical features of HD, including dystonia and chorea. Our team used CRISPR/Cas9 gene editing technology to precisely insert the human *mHTT* (150 CAG repeats in exon 1 of the HD gene) into the pig genome, and combined with somatic cell nuclear transfer technology, successfully established a gene knock-in (HD-KI) pig model of HD, which can simulate the typical pathological characteristics of selective death of medium-sized spiny neurons in the striatum of HD patients, and can also show “choreographic” behavioral abnormalities similar to HD in behavioral phenotypes 87. And these pathological characteristics and abnormal behaviors can be stably passed on to offspring.

**Figure 2:**
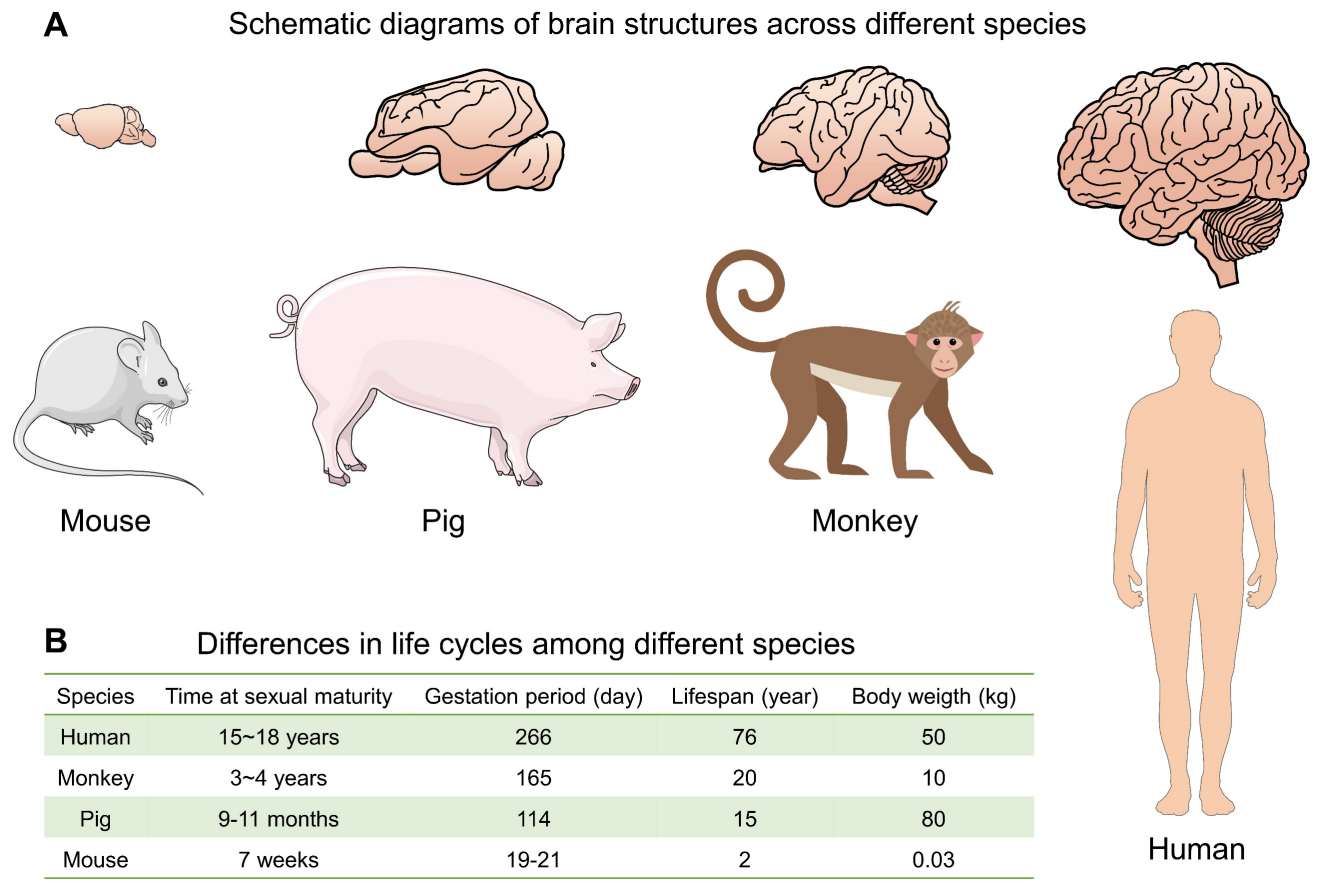
Compare the differences in brain structure and life cycle among different species.

Various disease models based on rodents have made important contributions to early basic research, but due to the great differences between humans and mice, the mouse model cannot fully simulate the clinical symptoms and pathological changes of patients, and many drug screenings fail in the later clinical transformation process 85. Therefore, animal models that can better simulate human diseases are needed for further evaluation. Compared with monkey models, pig models can give birth to multiple children in one litter, which is more suitable for research related to neurodevelopment. Miniature pigs have a short growth cycle and a fast multiplication rate, providing researchers with a rich material resource for studying neurodegenerative diseases using large animal models. Therefore, using large animal models to study the effects of mHTT on neurodevelopment from a new perspective of neurodevelopment has great potential (**Fig. 2**).

## 5. The potential of early developmental intervention to treat HD

For inherited genetic diseases, fetal gene therapy offers the ability to prevent early irreversible and lethal pathological changes. Although there have been no studies on the treatment of HD through embryonic or fetal periods, fetal treatment studies for neuropathic Gaucher disease (nGD) have been reported 88. nGD is caused by GBA mutations. In adult patients, milder manifestations are hepatomegaly, splenomegaly, and occasional lung and bone disease, which can be treated symptomatically with enzyme replacement therapy. However, the acute lethal form of nGD is untreatable. Intracranial injection of adeno-associated virus (AAV) vectors into the fetus reconstructed the expression of neuronal glucocerebrosidase. This therapy eliminated neurodegeneration and improved neuroinflammation, thereby significantly prolonging the survival of model mice. In addition, intervention in newborn mice also rescued mice, but the effect was poor.

In HD, there are numerous cases of changing developmental abnormalities or late disease progression through early intervention. For example, treatment with isoxazole-9, which targets key dysregulated pathways in neurodevelopmental processes, led to improvements in expanded polyglutamine repeat-related phenotypes in neurons, as well as improvements in cognitive impairment and synaptic pathology in HD model R6/2 mice 73. In addition, defects in neuroectoderm and rosette formation can be rescued by molecular and pharmacological approaches, thereby restoring the properties of the striatum 76. Similarly, mHTT in HD-hCOs disrupts the recruitment of ARF1 to the Golgi apparatus, altering the normal developmental trajectory, while restoring the recruitment of ARF1 to the Golgi apparatus in the HD neural tube can rescue altered corticogenesis in the HD fetal brain 78. Furthermore, HD forebrain organoids show abnormal neurodevelopment, and healthy cells in mosaic organoid tissues restore HD neurodevelopmental features, trajectories, synaptic density, and communication pathways at cell-cell contacts 79.

Our latest study shows that knocking out and repairing mutant genes in HD pig models using AAV adeno-associated virus vectors to express CRISPR/Cas9 gene editing, including stereotaxic brain injection and intravenous injection treatment, can effectively improve the neuropathology and behavior of newborn HD pigs or adult HD pigs 89. This study lays a theoretical foundation for early gene therapy of HD. In another study, Braz *et al.* observed that the excitatory synaptic activity of layer 2/3 of the cortex of HD model mice was low, the expression of glutamatergic receptors was low, and motor defects were shown in the first week after birth, while these defects could be self-repaired in the second week 65. Strengthening glutamatergic synaptic transmission with drugs in the neonatal period can rescue these neural defects and improve the behavioral disorders of HD model mice in adulthood. This not only proves the developmental defects of early HD, but also opens up ideas for the development of early drugs.

The above studies provide us with ideas for intervention and treatment of HD in the fetal period or neonatal period. HTT, as an important component of brain development, is indispensable for early neural development. mHTT seriously changes the neural development process of HD and plays a pivotal role in the occurrence and development of later HD. Therefore, intervening and treating HD at the beginning of development may be a therapeutic strategy to cure HD.

## 6. Conclusions and perspectives

To date, there have been many studies on the molecular and cellular mechanisms of abnormal development in HD, as well as studies on abnormal brain development in HD animal models. However, it was not until recent years that direct studies on how mHTT affects human neurodevelopment began. The classic concept is that HD is caused by the toxic mHTT protein acting on mature brain cells 90. However, there is increasing evidence to support another theory that mHTT affects animal brain development, and its effects may play an important role in neurodegeneration in adulthood. The ultimate pathological manifestation of HD is neurodegeneration, however, the process of its occurrence and development may be rooted in the pathophysiology of developmental abnormalities. Therefore, we systematically summarized the phenomena and mechanisms of mHTT affecting neuronal development in HD patients, animal models, and organoid models. This provides us with new ideas - understanding the pathophysiological mechanisms of HD from the perspective of neurodevelopment may be a completely new field. In this process, models that are more suitable for HD neurodevelopmental research will play a decisive role. This proposed the use of HD large animal models to explore the mechanisms of HD neurodevelopmental disorders, as well as therapeutic strategies for intervening in HD in the early stages of development to rescue disease progression.

In 2000, Mehler and Gokhan proposed that neurodegenerative diseases can be conceptualized as neurodevelopmental disorders, and their disease origins begin with abnormal development of the brain 91. Genes associated with neurodegenerative diseases are often expressed throughout neurodevelopment and are essential for the refinement and maintenance of neuronal subpopulations. Pathogenic mutations can impair certain neuronal functions in subtle ways, events that initially lead to dysregulation of cellular homeostasis in subsets of neurons in evolutionary domains, and adult-onset cell death in response to normal, nonlethal environmental stimulation 91. Thus, neurodegenerative diseases may represent an emerging class of developmental disorders characterized by novel biological responses to subthreshold neurodevelopmental abnormalities that are so subtle as to not cause overt developmental defects in targeted neuronal biosynthetic pathways, yet long-term compensatory efforts by cells to maintain normal function render them more susceptible to external insults.

Investigating the connection between neurodevelopmental abnormalities and neurodegeneration in HD may represent a crucial hub for understanding HD pathogenesis and holds significant implications for elucidating disease mechanisms and exploring novel therapeutic strategies. Abnormal neurodevelopmental phenomena have been demonstrated in both HD patients and HD models, yet these manifestations do not substantially impact normal daily life until the onset of HD symptoms. This leads us to question whether aberrant neurodevelopment genuinely influences HD disease progression. Notably, defective neural circuits have been identified in early postnatal HD mice, and rectifying these deficiencies can rescue later-stage HD symptoms 65. This compellingly demonstrates that neurodevelopmental abnormalities can indeed modify the process of neurodegeneration, and that early therapeutic interventions may ameliorate late-stage disease manifestations. It is evident that mHTT exerts profound effects on developmental processes, and how these alterations ultimately lead to late-stage neurodegeneration requires deeper investigation. Additionally, while current research predominantly focuses on the impact of HD on neuronal development, non-neuronal cells such as astrocytes, microglia, and oligodendrocytes play crucial roles in the progression of the disease. Therefore, investigating the effects of mHTT on the development of these non-neuronal cells and their influence on neurodevelopmental processes in HD represents a promising and underexplored area of research. In summary, neurodevelopmental defects establish the foundation for cellular vulnerability, while aging and accumulated cellular stress ultimately trigger the underlying vulnerabilities created by neurodevelopmental abnormalities, culminating in neurodegenerative progression.

Similarly, amyloid precursor protein (APP) plays an integral role in developmental processes 92-94. New research has found that APP may play a central role in the early stages of Alzheimer's disease, involving two finely regulated genetic mechanisms, including the canonical WNT signaling pathway that controls stem cell proliferation, and the activation of activator protein 1, which triggers the production of new neurons and thus regulates the timing of neurogenesis 95. Given the evidence, it seems imperative to draw the connection between development and neurodegeneration 96. In short, neurodegenerative diseases are no longer simply neurodegeneration, and the role of developmental factors in neurodegeneration is gradually revealed. This new theory will open up new avenues for studying new mechanisms and treatment strategies for neurodegenerative diseases.

## Figures and Tables

**Table 1:** List of evidence for neurodevelopmental abnormalities exhibited by human HD gene carriers.

Research subject	CAG number	Development stage	Developmental abnormalities	Reference
Children and adolescents (pre-HD)	42.78 ± 2.24Q	On average 15 years from disease onset	Cortical cell loss as measured by lower gray matter volume and higher mean gray matter diffusivity. Cortical cell loss was positively correlated with expression of developmental genes.	41
Children and adolescents (pre-HD)	47.58 ± 6.54Q	Children, adolescents and young adults	Significant deficits on a comprehensive measure of executive function and working memory occur before the onset of motor symptoms in HD.	47
Children and adolescents (pre-HD)	49±4.9Q (6-10 year with 46±5.2Q, 11-14 year with 44±5.3Q and 15-18 year with 42±3.9Q)	Children and adolescents (age 6-18)	GE initially presents with hypertrophy of the striatum and globus pallidus, with a more rapid rate of volume loss.	42
Children and adolescents (pre-HD)	51-73Q	8.64 ± 1.73 years	The mean striatal volume in presymptomatic HD children was significantly higher than that in normal children.	43
pre-HD	42.47±2.54Q	40.50±9.75 years	Atrophy of the caudate, putamen, globus pallidus, basal ganglia, and white matter was associated with subtle motor and cognitive measures early in the pre-HD phase.	37
pre-HD	43.1±2.4Q	40.8±8.9 years	Region-specific gray and white matter atrophy in presymptomatic HD.	38
Children at risk for HD with no manifest symptoms	45.15±5.05Q	13.96±3.43 years	Head circumference, weight, and BMI were significantly reduced.	35
pre-HD	42.1±1.8Q	36.7±8 years	preHD group showed striatal atrophy and increased clinical irritability ratings	45
Human HD fetal	39~46Q	Gestational weeks14~16	mHTT is mislocalized in the embryo, impairs endosomal secretion and recycling, disrupts the neuroepithelial junction complex, alters cell cycle progression, and biases neurogenesis toward the neuronal lineage	48
HD patients	46±4Q	42±8 years	There is no asymmetry in the length of the developmentally relevant sylvian fissure imprint	40

**Table 2:** List of neurodevelopmental abnormalities seen in HD animal models.

Research subject	CAG number	Development stage	Developmental abnormalities	Reference
Drosophila	UAS-127Q and UAS-Htt128Q	NA	Inhibition of cell cycle	93
Mouse	R6/2 (114Q)	Presymptomatic (5-7 weeks)	Large-amplitude synaptic events (>100 pA) occur more frequently	62
	R6/2 and R6/1 (115Q)	Adult	Hippocampal and olfactory bulb neurogenesis was reduced, and NeuroD1 expression was reduced in the hippocampus	56
	R6/2 (114Q)	Adult	Progenitor cells have an impaired migration	59
	ND:CRE-GFP + HTT-17Q or HTT-68Q	E14.5-E18.5	Pathological HTT loses its ability to promote neuronal migration	33
	BACHD:CAG-Cre (ERT2) mice (Q97CRE mice)	P21	These conditional knockout mice ultimately displayed injury signatures similar to those of mice that had lifelong expression of mHtt.	61
	HdhQ7/Q111	P1-P26	In early postnatal HD mice, excitatory synaptic activity was low and glutamatergic receptor expression was reduced in layer 2/3 of the cortex.	65, 66
	HdhQ111/Q111	E10.5, E14.5 and E16.5	mHTT causes mitotic spindle misorientation in dividing progenitors and reduces cortical thickness at embryonic days E14.5 and E16.5.	58
	Hdh-Q111	E12.5-E17.5	Delayed acquisition of early striatal cytoarchitecture, aberrant expression of progressive markers of MSN neurogenesis, delayed cell cycle exit of striatal progenitors, expansion of the intermediate progenitor pool, and overexpression of the transcription factor Sox2.	54
	HdhQ7/Q111	E15.5-P21	NUMA1 downregulation induces axonal growth defects in HD	64
	HdhQ7/Q111	E15.5-P21	The mHTT protein causes mitotic spindle misorientation and biases the fate of progenitor cells toward the neuronal lineage.	48
	YAC128	Mild (6month) and late (10month) symptomatic	Neural stem/progenitor cells derived from the YAC128 subventricular zone showed higher proliferation and migration capacity, with more MAP2+ and synaptophysin+ cells;NSPC from 10mo YAC128 mice exhibited lower mitochondrial membrane potential and increased mitochondrial Ca2+ accumulation	60
	zQ175 (polyQ stretch ranges between 175 and 200)	P21 and 5 week	There is excessive excitatory synapse formation and maturation in the cortex, which disappears after 5 weeks.	9
Rat	CAG51	P15 and P30	The average and radial diffusivity of white matter and gray matter structures in HD pups were higher and decreased with age, accompanied by an increase in fractional anisotropy in white matter structures	49
	CAG51	Adult	The olfactory bulb neurogenesis was significantly reduced	55

**Table 3:** List of HD iPSCs and iPSCs-derived organoid models for studying HD development.

Research subject	CAG number	Development stage	Developmental abnormalities	Reference
	60 and 109 repeats	day 56	Genes in glutamate and GABA signaling, axon guidance, and calcium influx were all reduced in HD	73
iPSCs lines from juvenile-onset HD	180, 109, or 77 CAG repeats	36, 60, 80, 100, and 130 days	HD iPSCs generated similar numbers of deep and upper layer cortical neurons, but HD cortical neurons exhibited delayed maturation and altered morphology, and increased CAG repeat length was directly correlated with decreased neurite length.	71
human cerebral organoids	WT/70Q, 70Q/70Q	d28 (for early neurogenesis), d49 (for late neurogenesis and astrogenesis)	Brain organoids carrying mHTT had reduced growth rate, significantly reduced "progenitors" and "proliferative progenitors" populations, downregulation of progenitor marker expression and neural development-related genes, and abnormal mitochondrial morphological dynamics of early neural cells.	77
cortical organoids	60Q, 109Q, 180Q	day8, 15, 30	HD causes complete failure of neural ectoderm acquisition, severe abnormalities in neural rosette formation, and abnormal gene expression profiles.	76
hiPSCs-derived neurons	HTTEx1Q72	d7, d14, d21, d30	Altered appearance of synaptic proteins and a reduction in synaptic contacts lead to a delay in the development of mature neuronal activity patterns.	72
HD cortical organoids (hCOs)	CAG 55 and 59	day 37, 40, 60	HD-hCO models exhibit deficient junctional complexes, premature neuronal differentiation, and delayed neuronal maturation, including the early formation of TBR2+ BPs, resulting in premature neuronal differentiation and the depletion of proliferation pools	78
telencephalic organoids	CAG 48, 56, 72	day35, 60, 120	neurodevelopmental abnormalities in ventral fate acquisition in HD telencephalic organoids, such as cytoarchitecture and transcriptional defects that result in a reduction in GABAergic neurons	79

## Abbreviations

AAV: adeno-associated virus
APP: amyloid precursor protein
CHCHD2: coiled-coil-helix-coiled-coil-helix domain containing 2
GE: gene-expanded
GNE: gene-nonexpanded
*Hdh*: homolog HD gene
HD: Huntington's disease
*HTT*: *huntingtin*
hCOs: human cortical organoids
iPSCs: induced pluripotent stem cells
polyQ: polyglutamine
*mHTT*: mutated *HTT*
nGD: neuropathic Gaucher disease
NSCs: neural stem cells
NPCs: neural progenitor cells
NSPCs: neural stem/progenitor cells
NUMA1: nuclear/mitotic apparatus protein 1
SVZ: subventricular zone
